# Increased Colonic Levels of CD8^+^ Cytotoxic T lymphocyte-Associated Mediators in Patients With Microscopic Colitis

**DOI:** 10.1093/ibd/izaf064

**Published:** 2025-04-10

**Authors:** Alexandra Lushnikova, Anna Wickbom, Johan Bohr, Robert Kruse, Anders Wirén, Elisabeth Hultgren Hörnquist

**Affiliations:** School of Medical Sciences, Örebro University, Örebro, Sweden; Division of Gastroenterology, Department of Medicine, Örebro University Hospital, Faculty of Medicine and Health, Örebro University, Örebro, Sweden; Division of Gastroenterology, Department of Medicine, Örebro University Hospital, Faculty of Medicine and Health, Örebro University, Örebro, Sweden; School of Medical Sciences, Örebro University, Örebro, Sweden; Department of Clinical Research Laboratory, Faculty of Medicine and Health, Örebro University, Örebro, Sweden; Clinical Epidemiology and Biostatistics, School of Medical Sciences, Faculty of Medicine and Health, Örebro University, Örebro, Sweden; School of Medical Sciences, Örebro University, Örebro, Sweden

**Keywords:** microscopic colitis, CD8^+^ T lymphocytes, colorectal cancer, ulcerative colitis, colonic biopsies

## Abstract

**Background:**

For unidentified reasons, possibly due to increased immune surveillance, patients with collagenous colitis (CC) and lymphocytic colitis (LC), both forms of microscopic colitis (MC), have lower risk of colorectal cancer than controls and ulcerative colitis (UC) patients. Levels of secreted and cell-bound mediators in MC patients with active disease and in histological remission (HR) compared to UC patients and controls were investigated.

**Methods:**

Median fluorescence intensity of 54 analytes in colonic biopsies from patients with active CC (*n* = 21), LC (*n* = 11), and UC (*n* = 19); CC-HR (*n* = 6), LC-HR (*n* = 9), UC in remission (*n* = 19), non-diarrhea controls (*n* = 48), and diarrhea controls (*n* = 25) was measured using Luminex.

**Results:**

Granzyme B and CCL5 levels were higher in active CC than in UC, whereas CCL4 and CD163 levels were similar in CC and UC, and both groups had higher levels of matrix metalloproteinase (MMP)-1, MMP-3, and tumor necrosis factor receptor II than both control groups. APRIL, BAFF, BCMA, CCL20, CXCL8, chitinase 3-like 1, pentraxin-3, Fas, and IL-33 were higher in UC than MC. Increases in 4-1BB and perforin in MC compared to controls were lower than in UC. Levels of gp130 and IL-6Rα were decreased in MC but increased in UC compared to controls.

**Conclusions:**

Microscopic colitis patients exhibit increased levels of several analytes, including some associated with CD8^+^ T lymphocytes, suggesting a different pathogenesis of MC compared to UC. Higher levels of MMP-1 and MMP-3 in CC than LC indicate separate disease entities.

## Graphical Abstract

**Graphical Abstract:**
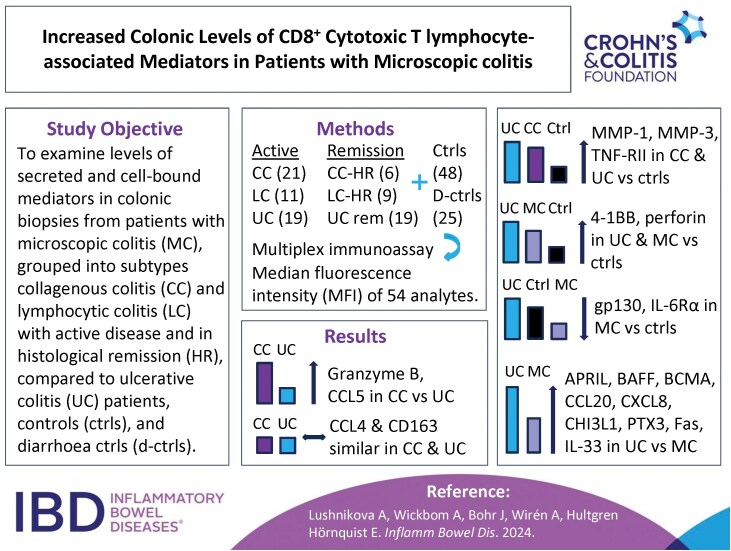

**Key Messages**

**What is already known?**

- Microscopic colitis (MC) patients have increased numbers of CD8^+^ T lymphocytes that are involved in immune surveillance. They also have a decreased risk of developing colorectal cancer compared to ulcerative colitis (UC) patients and controls.

**What is new here?**

- Colonic levels of several immune mediators, some that were studied for the first time in MC patients, and some being associated with CD8^+^ T lymphocytes, were found to be increased in patients with MC compared to controls and, in some cases, UC patients.

**How can this study help patient care?**

- These findings reveal immunological differences between MC and UC and between the 2 subtypes of MC, lymphocytic colitis and collagenous colitis.

## Introduction

Microscopic colitis (MC) is an inflammatory bowel condition with symptoms including watery, non-bloody diarrhea, weight loss, and abdominal pain.^1^ Microscopic colitis is divided into 2 subtypes: lymphocytic colitis (LC) and collagenous colitis (CC). Microscopic colitis is most prominent in older females and one study reports an incidence of 14.2 cases per 100 000 person-years.^2^ The pathogenesis of MC is not yet fully known but, in genetically predisposed individuals, a dysregulated immune response may be invoked following exposure to certain components in the intestinal lumen.^1^ Endoscopic findings in patients with MC are macroscopically mostly normal, but histologic examination of colonic biopsies reveals abnormalities such as a thickened subepithelial collagen layer (>10 μm) in patients with CC and increased numbers of intraepithelial lymphocytes (IELs; >20 per 100 epithelial cells) in patients with LC.^1^ A diagnosis of incomplete MC (MCi) is sometimes made if patients show typical signs of MC but fail to completely meet the diagnostic criteria.^1^

Chronic colonic inflammation in patients with ulcerative colitis (UC) and Crohn’s disease (CD), both forms of inflammatory bowel disease (IBD), is a risk factor for the development of colorectal cancer (CRC),^3^ yet patients with MC paradoxically do not have an increased risk of developing CRC^4^ and, for unknown reasons, instead display a decreased risk compared to matched reference individuals.^5–7^ However, some studies have found an increase in lymphoma^6^ and lung cancer^4,6^ in patients with MC, indicating that local factors affect the decreased risk of CRC. One protective mechanism against tumor development is the detection and elimination of malignantly transformed cells, a process called immune surveillance in which CD8^+^ T cells participate.^8^ We have previously demonstrated higher proportions of CD8^+^ IELs in LC patients compared to controls, CC, and UC patients.^9^ Furthermore, we found decreased numbers of CD4^+^ IELs in CC patients compared to controls and, in the lamina propria, CD4^+^ lymphocytes were decreased in LC and CC compared to controls.^10^ In contrast, in IBD, the differences in T cell subsets in comparison to controls were decreases in CD8^+^ and increases in CD4^+^ T cells.^11^ Another positive contributor to immune surveillance may be the augmented numbers of IELs observed in MC.^7^ If patients with MC possess enhanced capabilities to utilize this process, it may partially explain their decreased risk of CRC.

Through the release of cytotoxic molecules, such as perforin and granzyme B, as well as the interaction of Fas with Fas ligand (FasL), CD8^+^ T cells can exert many detrimental effects on tumor cells.^8^ There is limited information regarding these cytotoxic molecules and the possible roles of CD8^+^ T cells in MC, hence further research is necessary. By investigating a wide range of secreted and cell-bound mediators such as chemokines and cytokines, among others, in colonic biopsies from patients with MC, UC, and non-inflamed controls, this study aims to increase knowledge about the local immune response during colonic inflammation in patients with MC compared to UC and non-inflamed controls.

## Materials and Methods

### Patient Demographics and Biopsy Collection

In total, 158 samples from 156 patients were examined (Table 1). Histological remission denotes patients with a previous histopathologically confirmed diagnosis of MC that now presented with clinical symptoms, but histological examination of the colonic biopsy did not fulfill the criteria for MC diagnosis. Patients with MC and UC were diagnosed according to current diagnostic guidelines.^1,12^ In patients with UC, the mean Mayo endoscopic subscore (on a scale of 0 to 3) was 1.7 (SD 0.7) in those with active disease and 0.1 (SD 0.2) in patients in remission.

**Table 1.** Patient demographics including immunomodulatory treatment.

Diagnosis	Sex: *n* (%)		Mean age (SD)	Immunomodulatory treatment	
	Male	Female		*N* (%)	Medication(s) (number of patients)
Non-diarrhea control (*n* = 48)	24 (50)	24 (50)	58.7 (17.0)	5 (10)	Betamethasone (1), methotrexate (1), methotrexate, prednisolone, and sulfasalazine (1), prednisolone (1), methotrexate and diclofenac (1)
Diarrhea control (*n =* 25)	6 (24)	19 (76)	49.3 (18.2)	0 (0)	N/A
LC active (*n *= 11)	0 (0)	11 (100)	68.7 (9.3)	1 (9)	Budesonide (1)
CC active (*n *= 21)	3 (14)	18 (86)	60.3 (13.1)	5 (24)	Budesonide (4), cyclosporine and prednisolone (1)
UC active (*n* = 19)	13 (68)	6 (32)	50.1 (15.7)	14 (74)	Azathioprine (1), mesalazine (11), mesalazine and methotrexate (1), olsalazine (1)
LC-HR (*n *= 9)	0 (0)	9 (100)	57.0 (22.8)	1 (11)	Budesonide (1)
CC-HR (*n *= 6)	0 (0)	6 (100)	60.2 (9.0)	2 (33)	Budesonide (1), budesonide and sulfasalazine (1)
UC remission (*n* = 19)	13 (68)	6 (32)	56.0 (13.6)	12 (63)	Balsalazide (1), mesalazine (4), olsalazine (4), sulfasalazine (3)

Samples from one patient with LC were included both when in active disease and when in HR and samples from one patient with active UC were included twice with an interval of approximately a year between biopsies being taken. Information about immunomodulatory treatment was unavailable for one control.

Abbreviations: CC, collagenous colitis; CC-HR, collagenous colitis in histological remission; LC, lymphocytic colitis; LC-HR, lymphocytic colitis in histological remission; UC, ulcerative colitis.

Exclusion criteria were earlier diagnosis of CD and/or clinical symptoms of gastrointestinal infection, ischemic colitis, or neoplastic disease. Controls were separated into 2 groups, those with and those without diarrhea. Five non-diarrhea controls received immunomodulatory treatment due to asthma, polymyalgia rheumatica, psoriatic arthritis, or rheumatoid arthritis and information about immunomodulatory treatment was unavailable for one control (Table 1). Both control groups displayed no signs of colonic inflammation and underwent colonoscopy for further investigation of clinical symptoms.

Patient records of the diarrhea controls showed the presence of concomitant irritable bowel syndrome (IBS) at biopsy (*n* = 1) and the absence of IBS (*n* = 2), with information being unavailable for the remaining 22 diarrhea controls. The frequency of diarrhea ranged from 1 to 10 episodes per day in the 9 diarrhea controls for which information was available. There was no information regarding the presence of incomplete microscopic colitis in diarrhea controls.

A standard forceps was used to obtain, on average, 3 biopsies from the hepatic flexure in both control groups and MC patients, and from the macroscopically affected parts of the (distal) colon in UC patients. All biopsies were collected at Örebro University Hospital, Sweden, and stored at −80 °C in RNAlater (Ambion, Inc.) prior to use.

### Protein Lysate Preparation

Preparation of protein lysates was performed using a TissueLyser LT (Qiagen) and the Pierce BCA Protein Assay Kit (Cat. #23227, Thermo Fisher Scientific), as described previously.^13^

### Luminex Analysis

Protein lysates were analyzed at a total protein concentration of 2 mg/mL, apart from granzyme A for which analysis was performed at a total protein concentration of 0.05 mg/mL. The median fluorescence intensity (MFI) was used for analysis rather than absolute protein concentrations to allow inclusion of samples with concentrations below the lowest standard that would otherwise have to be dealt with by, for example, imputation.^14,15^ The median fluorescence intensity without subtraction of blanks was used, as suggested in a previous study.^14^ The Luminex® 200 Instrument System, together with xPONENT 3.1 software, was used.

In total, 54 analytes were investigated. The Human Luminex Discovery Assay (Cat. #LXSAHM, R&D Systems) was used in 5 of the 7 different panels (Table 2) consisting of 2 plates, apart from panel 4 which consisted of 3 plates. The Milliplex Map Human CD8^+^ T cell Magnetic Bead Panel – Immunology Multiplex Assay (Cat. #HCD8MAG-15K, Merck Millipore) was used in the remaining 2 panels (6-7) (Table 2). The assays were customized to analyze a combination of different analytes according to the manufacturers’ recommendations. Most often, at least one sample from each plate was also run on another plate from the same kit to assess interplate variation. A previous study has shown that interplate variation is decreased when MFI is used instead of absolute protein concentrations.^15^

**Table 2.** All 54 analytes grouped by the panel the analytes were analyzed in.

Panel	Analytes
1	**CXCL8 (IL-8)**, IL-11, IL-12/IL-23 p40, IL-19, IL-27, IL-34, **IL-6Rα**, **gp130**, IFN-β **MMP-1**, **MMP-3** **Chitinase 3-like 1**, **CD163**, **pentraxin-3**, **APRIL**, **BAFF**, osteopontin, aggrecan
2	IL-2, IL-4, IL-5, IL-6, IL-10, IL-13 CCL3 (MIP-1α), **CCL4 (MIP-1β)** GM-CSF, TNF-α, IFN-g, **4-1BB** **Fas**, Fas Ligand, **granzyme B**
3	IL-1β, IL-12 p70, IL-15, IL-17 (IL-17A), IL-17E (IL-25), IL-21, IL-23, IL-28A, IL-31, **IL-33** **CCL20 (MIP-3α)**, CD40 Ligand, TNF-β
4	CD30, TNF-RI, **TNF-RII**
5	**CCL5 (RANTES)**, **BCMA**, **MMP-2**
6	**Perforin**
7	**Granzyme A**

Bold text indicates the 22 analytes with a sufficiently high fold increase in MFI values to be included in the statistical analysis.

Abbreviations: APRIL, a proliferation-inducing ligand; BAFF, B-cell activating factor; BCMA, B-cell maturation antigen; IFN-β, interferon-β; IL, interleukin; GM-CSF, granulocyte macrophage colony-stimulating factor; MMP-1, matrix metalloproteinase-1; TNF-RII, tumor necrosis factor receptor II.

Due to some samples having bead counts below a recommended cutoff of 30, or having insufficient amounts of total protein, not all samples were included in each assay. The number of samples investigated in each group for each analyte is shown in the Figures in the Results section.

### Statistical Analysis

To discern factors that might confound the investigated associations between analyte levels and diagnosis, non-metric multidimensional scaling (MDS) analysis, similar to principal component analysis, was performed based on all 54 analytes in R version 4.3.1^16^ using the MASS package.^17^ Graphs were inspected for clustering based on sex, age group, Luminex plate, and diagnosis. The 5 age groups, in years, were 19-41 (*n *= 30), 42-52 (*n* = 31), 54-63 (*n* = 34), 64-71 (*n* = 30), and 72-88 (*n* = 33). Missing values were imputed with the mean of the affected diagnosis group, and missingness was ≤10% for most analytes apart from matrix metalloproteinase (MMP)-2 (18%) and perforin (31%). The values were first scaled, followed by log10 transformation. Spearman’s correlation coefficient was used to create the correlation matrix, after which a distance matrix was created. Imputation, scaling, and transformation were only performed for the purpose of MDS analysis. All other analyses were performed on the original scale and with no imputed values. In addition to inspecting the graphs for clustering, MDS was also performed as an evaluation step to provide information about multivariable patterns with potential for machine learning classification.

To decide which of the 54 analytes had a sufficiently high signal-to-noise ratio to be included in the statistical analysis, a ratio was calculated for each analyte by dividing the median MFI value of each diagnosis group by the mean MFI value of the blanks per plate. The diagnosis group with the highest ratio was selected and the range spanned from 1038.7 to 0.8. Based on an observed break point at an analyte/blank MFI ratio of 5.7, a cutoff ratio of 5.7 was chosen. Twenty-two analytes had a ratio ≥ 5.7 and the remaining analytes had a ratio below 4.8. An additional MDS analysis was performed on the 22 analytes with a ratio ≥ 5.7. Spearman’s correlation was performed to determine whether age was correlated with analyte levels.

For each of the 22 selected analytes, further investigation of possible confounding of results due to sex and plate was performed. Boxplots of analyte level grouped by sex and plate, as well as scatterplots grouped by diagnosis and separated by sex and plate were inspected. Associations between analyte levels and sex, diagnosis and sex, analyte levels and plate, and diagnosis and plate were estimated by Kruskal-Wallis, Chi-squared, and Mann-Whitney U tests, with adjustment for multiple comparisons using the Benjamini-Hochberg approach.

A Kruskal-Wallis test was performed on the MFI values for each of the remaining 22 analytes in GraphPad Prism version 10.3.1 for Windows (GraphPad Software, www.graphpad.com). P-values from the 22 tests were adjusted for multiple comparisons in R version 4.3.1^16^ using the Benjamini-Hochberg approach.^18^ For analytes that showed a significant adjusted *P*-value, a Dunn’s post hoc test was performed in GraphPad Prism to ascertain which diagnosis groups had different levels of the respective analyte. Two post hoc comparisons deemed to be irrelevant (collagenous colitis in histological remission [CC-HR] vs LC active and lymphocytic colitis in histological remission [LC-HR] vs CC active) were excluded. A confidence level of 0.05 was used. Outliers were detected using the Dixon-Reed method^19^ using a custom script in R version 4.3.1.^16^

### Multivariable Evaluation With Supervised Machine Learning Methods

To increase the number of individuals in training and testing during machine learning, both control groups (controls with and without diarrhea) were combined into one group (*n* = 72), and MC patients with LC and CC were combined into one group (MC), and all MC patients (with active disease and in remission) were also combined as another group (MC (all); *n* = 34). Machine learning was performed as randomized nested cross-validation. Within each randomized nest, the data were partitioned into training (80%) and testing (20%) sets. Each set of data was standardized followed by bagged tree model imputation of missing data (recipe package in R) and class balancing with Adaptive Synthetic Algorithm minority over-sampling technique (Themis package in R) of the training set. The number of proteins of the training set was within training reduced by Boruta selection with a cutoff of 0.01 (colino package in R). Thereafter, regularized logistic regressions (LRs; glmnet package in R), Random forests (RFs; ranger package in R), XGBoosted trees (XG; XGboost package in R), Neural network (NN; nnet package in R), Naïve Bayes (NB; klaR package in R), Radial basis support vector machine (SVM; discrim package in R) and lightGBM (LGBM; bonsai package in R), and Stacked model of LR, RF, and NN (ST; stacks package in R) were in parallel trained with hyperparameter tuning. Hyperparameters for LR (mixture and penalty), RF (number of variables included in each random tree and minimum n for split), XG (number of variables included in each tree, tree depth, loss reduction, learning rate, and minimum n for split), NN (number of hidden layers and penalty), NB (smoothness and laplace), SVM (cost and sigma), LGBM (number of variables included in each tree, tree depth, loss reduction, and learn rate), and ST (hyperparameters of LR, RF, and NN) were tuned with a Latin Hypercube search approach with internal validation on 25 bootstraps of the training data with classification performance evaluation scored as the area under the curve (AUC). Optimal hyperparameters were used in the final models, and performance was assessed on the hold-out testing data of each nest. To estimate the robustness of predictions with regards to random effects of initial partitioning, this workflow procedure was repeated iteratively 30 times as randomized nested cross-validation with random partitioning of samples to training and testing set at each iteration. The variability of model performances from the nests was estimated by fitting Bayesian models and Markov Chain Monte Carlo via the tidyposterior and rstanarm packages in R, with 5000 iterations, 4 chains, and a prior normal distribution for the random (nest) intercepts. Posterior probability distributions of mean AUC and their contrasted differences between all models were evaluated for practical equivalence. Model variable importance scores (VIP packages in R) with coefficient values were obtained from the final models of each nest.

## Results

### Increased Levels of Granzyme B, CCL5, and Granzyme A in MC Compared to UC Patients

**Figure 1.**
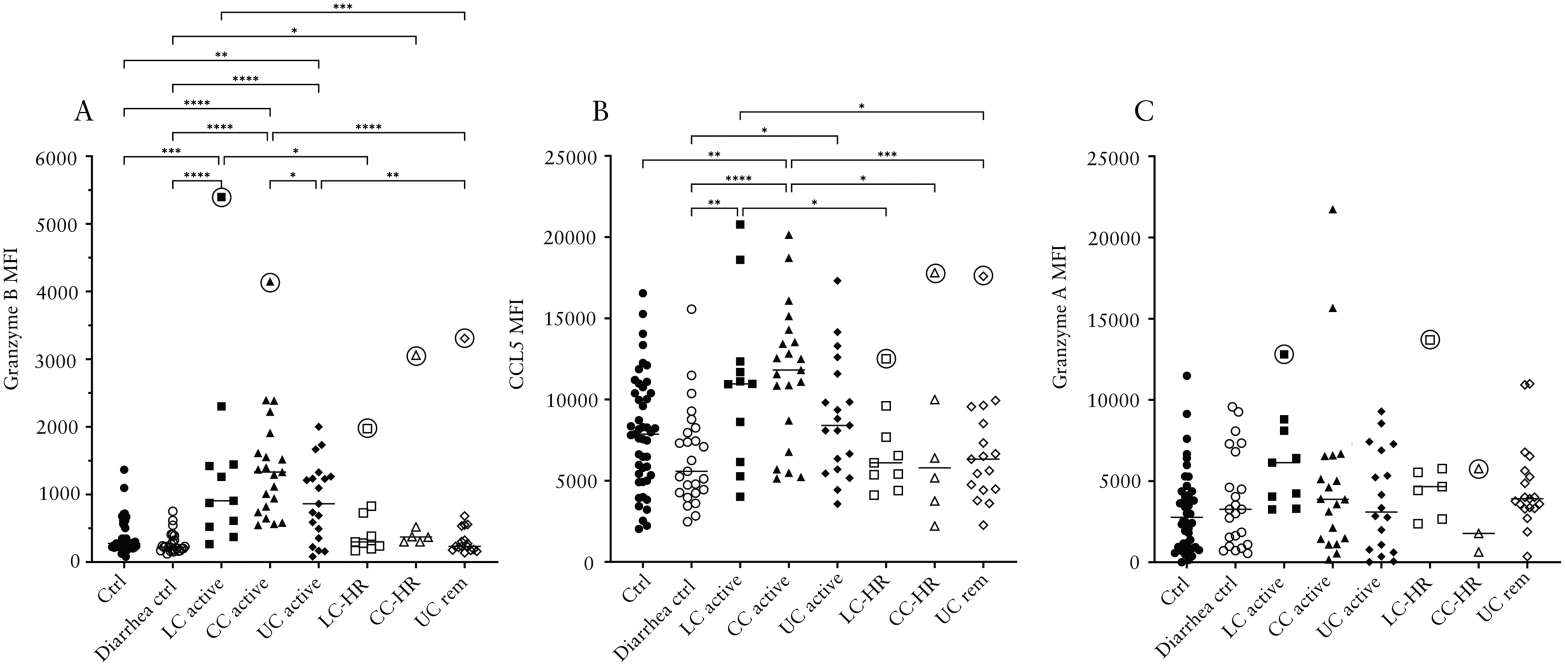
Median fluorescence intensity (MFI) of granzyme B (A), CCL5 (B), and granzyme A (C) in patients with active lymphocytic colitis (LC) and LC in histological remission (LC-HR), active collagenous colitis (CC) and CC in histological remission (CC-HR), active ulcerative colitis (UC) and UC in remission (UC rem), non-diarrhea controls, and diarrhea controls. Each point represents one patient, and the horizontal line marks the median of each group. Outliers found using the Dixon-Reed method are encircled. *P ≤ .05, **P ≤ .01, ***P ≤ .001, ****P ≤ .0001.

In patients with active CC, levels of the cytotoxic serine protease granzyme B were significantly increased compared to patients with active UC, whereas levels in active LC patients were comparable to active UC patients (Figure 1A). In active LC, CC, and UC, levels were significantly increased compared to both control groups and patients with UC in remission (Figure 1A). In all disease groups, levels were higher in patients with active disease compared to patients in remission, with this being statistically significant for LC and UC patients (Figure 1A).

Likewise, levels of the chemokine CCL5 (RANTES) which is stored in and degranulated from activated CD8^+^ T cells^20^ were higher in patients with active CC and LC compared to patients with active UC, although this did not reach statistical significance (*P* = .072 and *P* = .353, respectively). Patients with active CC had significantly increased levels in comparison to both control groups and CC and UC patients in remission, and patients with active LC had significantly raised levels compared to diarrhea controls, as well as LC and UC patients in remission (Figure 1B).

The cytotoxic protease granzyme A showed the highest levels in patients with active LC, followed by LC-HR although this failed to reach statistical significance following a Kruskal-Wallis test. The granzyme A levels in the remaining disease groups were not different from either of the control groups (Figure 1C).

### Similarly Enhanced Levels of CCL4 and CD163 in CC and UC Patients

**Figure 2.**
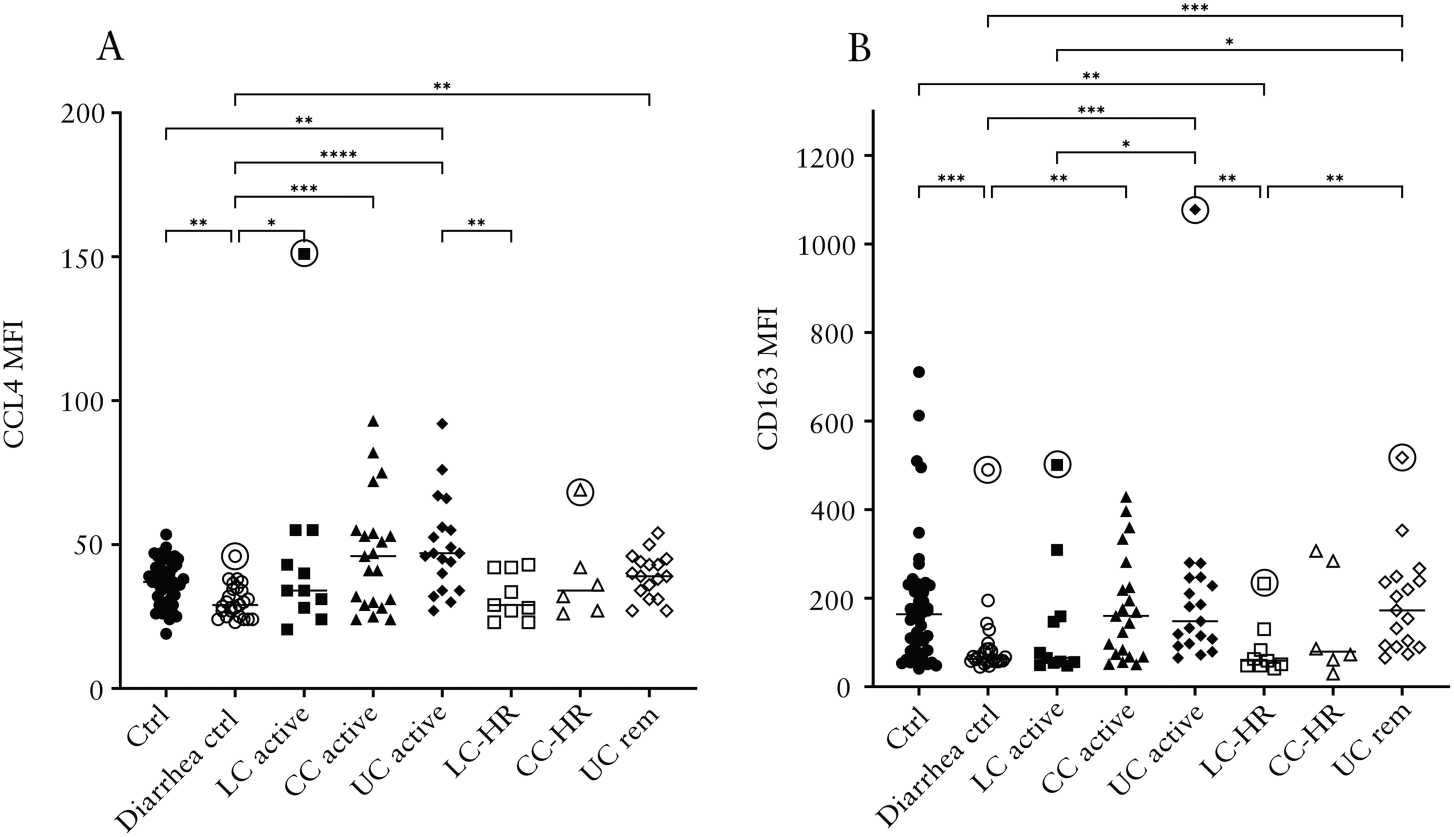
Median fluorescence intensity (MFI) of CCL4 (A) and CD163 (B) in patients with active lymphocytic colitis (LC) and LC in histological remission (LC-HR), active collagenous colitis (CC) and CC in histological remission (CC-HR), active ulcerative colitis (UC) and UC in remission (UC rem), non-diarrhea controls, and diarrhea controls. Each point represents one patient, and the horizontal line marks the median of each group. Outliers found using the Dixon-Reed method are encircled. *P ≤ .05, **P ≤ .01, ***P ≤ .001, ****P ≤ .0001.

Levels of CCL4 (MIP-1β), a pro-inflammatory chemokine, were significantly raised in patients with active CC and LC compared to diarrhea controls (Figure 2A). Patients with active UC displayed significantly increased levels compared to both control groups (Figure 2A).

Levels of the macrophage marker and scavenger receptor CD163 were similar to non-diarrhea controls in patients with active CC, active UC, and UC in remission (Figure 2B). In contrast, patients with LC-HR had significantly decreased levels compared to non-diarrhea controls and CC-HR also followed this trend, although not statistically significant (Figure 2B). Thus, whereas patients with active CC, active UC, or UC in remission had CD163 levels not different from non-diarrhea controls, patients with active LC, LC-HR, or CC-HR had reduced levels compared to non-diarrhea controls, but comparable to diarrhea controls.

### Increased Levels of 4-1BB and Perforin in Active LC, CC, and UC Patients

**Figure 3.**
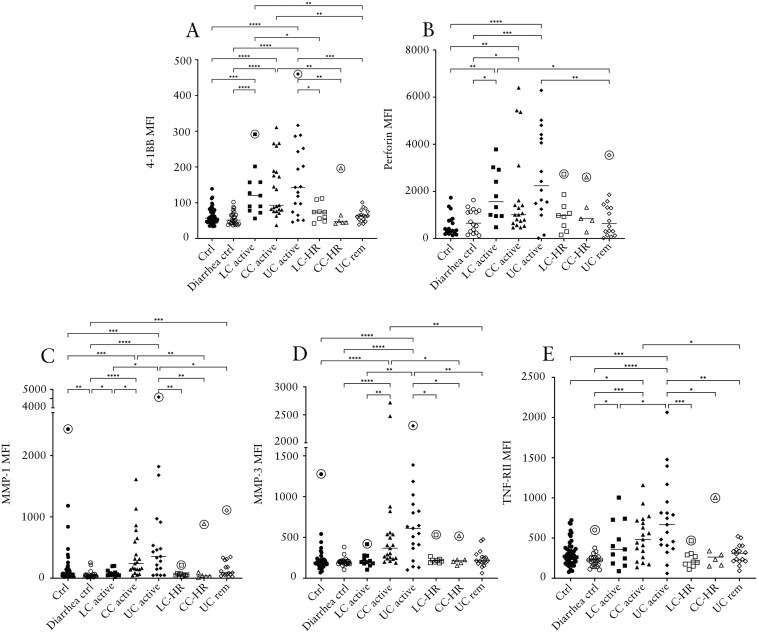
Median fluorescence intensity (MFI) of 4-1BB (A), perforin (B), MMP-1 (C), MMP-3 (D), and TNF-RII (E) in patients with active lymphocytic colitis (LC) and LC in histological remission (LC-HR), active collagenous colitis (CC) and CC in histological remission (CC-HR), active ulcerative colitis (UC) and UC in remission (UC rem), non-diarrhea controls, and diarrhea controls. Each point represents one patient, and the horizontal line marks the median of each group. Outliers found using the Dixon-Reed method are encircled. *P ≤ .05, **P ≤ .01, ***P ≤ .001, ****P ≤ .0001. MMP, matrix metalloproteinase; TNF-RII, tumor necrosis factor receptor II.

Significant increases in levels of the co-stimulatory receptor 4-1BB and the cytotoxic pro-apoptotic protein perforin were seen in patients with active LC, CC, and UC compared to both control groups (Figure 3A and B). The highest levels were seen in patients with active UC, followed by active LC and then CC, albeit with no significant differences between the 3 groups (Figure 3A and B). All 3 groups displayed significantly higher 4-1BB levels compared to UC patients in remission, and patients with active LC or CC had significantly higher levels than their counterparts in histological remission (Figure 3A).

For perforin, patients with active LC and UC showed significantly increased levels compared to UC in remission (Figure 3B).

### Higher Levels of MMP-1, MMP-3, and Tumor Necrosis Factor Receptor II in CC and UC Patients

Levels of the metalloproteinases MMP-1 and MMP-3 were highest in patients with active UC, followed by patients with active CC (Figure 3C and D). Patients with active CC had significantly increased levels compared to both control groups and patients with active LC, whereas patients with active UC had significantly higher levels compared to all other groups except patients with active CC (Figure 3C and D). Patients with active LC had significantly enhanced levels of MMP-1 but not MMP-3 compared to diarrhea controls but not non-diarrhea controls (Figure 3C and D).

In contrast, no significant differences in the levels of MMP-2 were observed between the different diagnosis groups (data not shown).

Levels of tumor necrosis factor receptor II (TNF-RII) were significantly increased in patients with active CC and active UC compared to both control groups, whereas in patients with active LC only compared to diarrhea controls (Figure 3E). Tumor necrosis factor receptor II levels were also significantly higher in patients with active CC or UC compared to UC in remission (Figure 3E).

### More Prominent Increases of APRIL, BAFF, BCMA, CCL20, CXCL8, Chitinase 3-Like 1, Pentraxin-3, Fas, and IL-33 in UC Than in LC and CC

**Figure 4.**
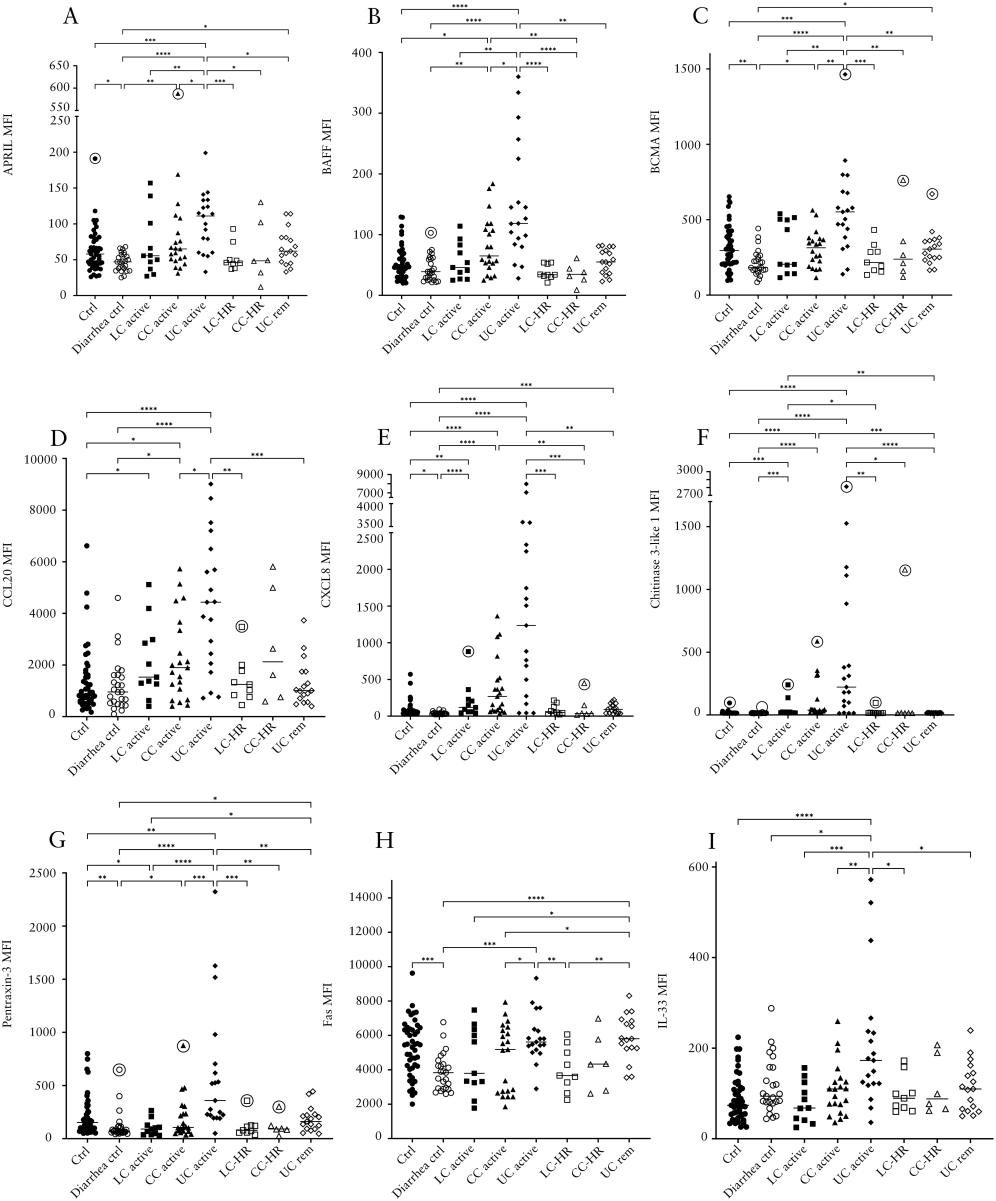
Median fluorescence intensity (MFI) of APRIL (A), BAFF (B), BCMA (C), CCL20 (D), CXCL8 (E), chitinase 3-like 1 (F), pentraxin-3 (G), Fas (H), and IL-33 (I) in patients with active lymphocytic colitis (LC) and LC in histological remission (LC-HR), active collagenous colitis (CC) and CC in histological remission (CC-HR), active ulcerative colitis (UC) and UC in remission (UC rem), non-diarrhea controls, and diarrhea controls. Each point represents one patient, and the horizontal line marks the median of each group. Outliers found using the Dixon-Reed method are encircled. *P ≤ .05, **P ≤ .01, ***P ≤ .001, ****P ≤ .0001. APRIL, a proliferation-inducing ligand; BAFF, B-cell activating factor; BCMA, B-cell maturation antigen; IL-33, interleukin-33.

For the analytes APRIL (a proliferation-inducing ligand), BAFF (B-cell activating factor), and BCMA (B-cell maturation antigen), all involved in B-cell maturation, significantly higher levels were found in patients with UC compared to patients with active CC or LC (Figure 4A-C). Levels of BAFF were significantly raised in patients with active CC compared to both control groups, whereas APRIL and BCMA were only increased compared to diarrhea controls (Figure 4A-C). Patients with active UC had significantly increased levels of APRIL, BAFF, and BCMA compared to all other groups (Figure 4A-C).

Levels of the chemokines CCL20 (MIP-3α) and CXCL8 (interleukin [IL]-8) were significantly increased in patients with active CC and UC compared to both control groups (Figure 4D and E). Patients with active LC showed significantly increased levels of CXCL8 compared to both control groups, whereas levels of CCL20 were increased in active LC patients only compared to non-diarrhea controls (Figure 4D and E). Levels of CCL20 were significantly increased in patients with active UC (*n* = 19) compared to active CC (*n* = 21) but no statistically significant differences in CCL20 or CXCL8 between patients with active UC and active LC (*n* = 11) were observed (Figure 4D and E).

Chitinase 3-like 1 was significantly increased in patients with active LC, CC, and UC compared to both control groups with the increases in LC and CC of a smaller magnitude compared to those in UC, albeit there was no significant difference between UC, CC, and LC (Figure 4F).

Pentraxin-3 levels were significantly higher in UC patients compared to patients with active CC or LC in addition to all the other groups, although active CC patients had significantly increased levels compared to diarrhea controls (Figure 4G). Levels in patients with active LC were significantly lower than in non-diarrhea controls (Figure 4G).

The median levels of the apoptosis-inducing cell-surface receptor Fas were significantly decreased in patients with active CC compared to active UC and UC in remission, as well as in active LC compared to UC in remission (Figure 4H). Finally, IL-33, a cytokine with both pro-inflammatory and regulatory roles, displayed significantly increased levels only in patients with active UC (Figure 4I).

For CXCL8, diarrhea controls and CC-HR patients had a lower median MFI value than the mean MFI of the lowest standard, and this was also true for chitinase 3-like 1 for both control groups, as well as patients with LC-HR, CC-HR, and UC in remission. Thus, comparisons between these groups should be interpreted with some caution due to uncertainties in the measurements of the low levels of these analytes.

### Decreased Levels of IL-6Rα in Both LC and CC and gp130 in LC Compared to Non-Diarrhea Controls

**Figure 5.**
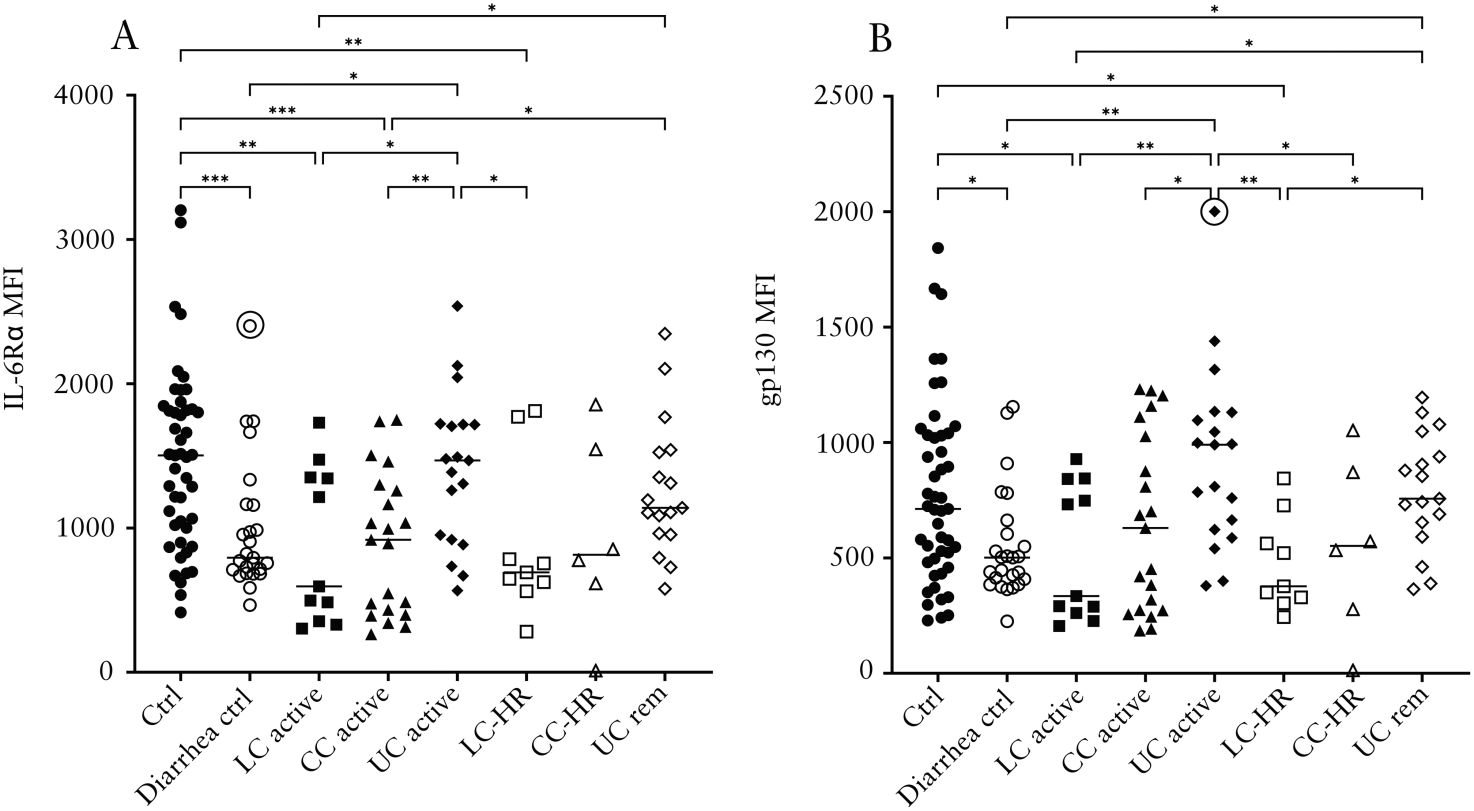
Median fluorescence intensity (MFI) of IL-6Rα (A) and gp130 (B) in patients with active lymphocytic colitis (LC) and LC in histological remission (LC-HR), active collagenous colitis (CC) and CC in histological remission (CC-HR), active ulcerative colitis (UC) and UC in remission (UC rem), non-diarrhea controls, and diarrhea controls. Each point represents one patient, and the horizontal line marks the median of each group. Outliers found using the Dixon-Reed method are encircled. *P ≤ .05, **P ≤ .01, ***P ≤ .001.

In contrast to the other mediators investigated, levels of IL-6Rα and its receptor subunit gp130 were significantly decreased in patients with active LC and LC-HR compared to non-diarrhea controls (Figure 5A and B). Patients with active CC also had significantly decreased levels of IL-6Rα compared to non-diarrhea controls (Figure 5A). The opposite was seen in patients with active UC, having significantly increased levels of IL-6Rα and gp130 compared to patients with active LC or CC and diarrhea controls (Figure 5A and B). Patients with UC in remission also showed significantly higher levels of IL-6Rα compared to patients with active CC or LC, and gp130 compared to patients with active LC, LC-HR, and diarrhea controls. (Figure 5A and B).

Additionally, increased levels of the pro-inflammatory cytokines IL-1β and IL-17 in UC patients with active disease compared to all other groups were observed. However, IL-1β and IL-17, with median analyte/blank MFI ratios of 4.8 and 4.7 respectively, failed to meet the set cutoff criteria of ≥ 5.7 and were therefore excluded from statistical analysis.

The remaining analytes, IL-2, IL-4, IL-5, IL-6, IL-10, IL-11, IL-12/IL-23 p40, IL-12 p70, IL-13, IL-15, IL-17E, IL-19, IL-21, IL-23, IL-27, IL-28A, IL-31, IL-34, TNF-α, TNF-β, interferon (IFN)-β, IFN-Ɣ, CCL3, FasL, granulocyte macrophage colony-stimulating factor (GM-CSF), osteopontin, aggrecan, CD40 ligand, CD30, TNF-RI had even lower analyte/blank MFI ratios and were likewise excluded from statistical analysis.

### Few Signs of Confounding by Sex, Plate, or Age

Neither MDS performed on all 54 analytes nor on the 22 analytes with an analyte/blank MFI ratio ≥ 5.7 showed clustering by sex, plate, or age group and thus no indication of confounding by these factors was seen, whereas a slight clustering by diagnosis was observed and further evaluated with machine learning classification (Figures S1 and S2). The final stress values achieved for MDS performed on all 54 analytes and the 22 analytes were 0.086 and 0.122, respectively. Spearman’s correlation failed to show a correlation between analyte levels and age (Table S1).

At the level of each individual analyte, no signs of confounding by plate were seen. Possible confounding due to sex was observed for BCMA, where males had higher levels than females. Graphs and data for these analyses are available on request.

### Multivariable Machine Learning Classification of MC Patients

**Figure 6.**
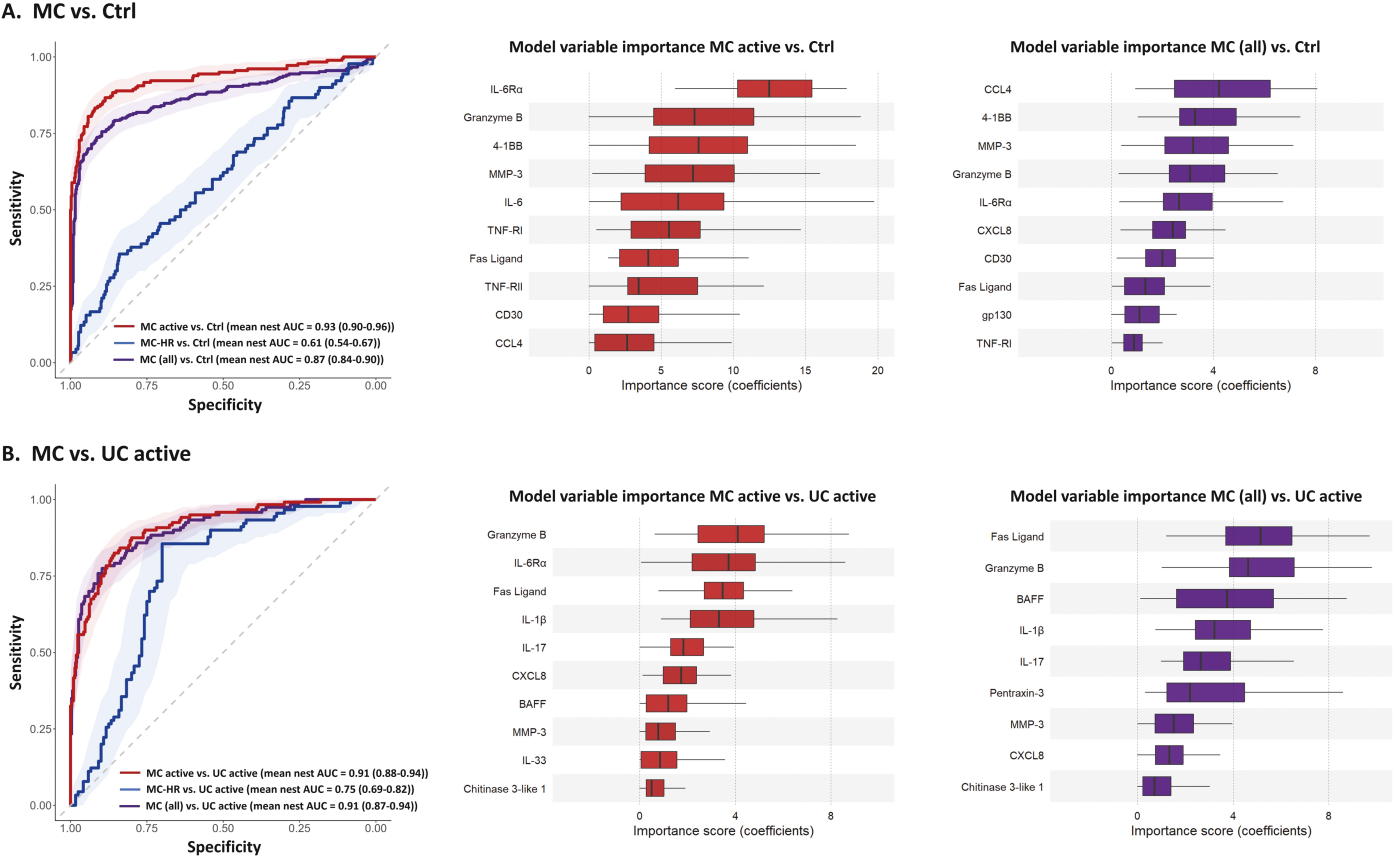
Performance of protein levels for multivariable classification of MC. Machine learning classification, performed by penalized logistic regression in 30 nested cross-validations, and their corresponding model variable importance scores for (A); MC active, MC-HR, and all MC vs. controls, and (B); MC active, MC-HR, and all MC vs. UC active. The left section shows the averaged receiver operating characteristic (ROC) curve of nests with 95% CI (mean area under the curve [AUC] and 95% CI shown in legend parentheses) for predictions of MC. The boxplots show the model importance scores of the top-10 proteins selected by at least 80% of model fits in the nested cross-validations for (A); MC active and all MC vs. controls and (B); MC active and all MC vs. UC active. The left and right borders of the boxes indicate the first and third quartiles, the lines in the middle represent the median, and the whiskers extending to the most extreme points within 1.5 times the interquartile range (IQR).MC, microscopic colitis; HR, histological remission; UC, ulcerative colitis.

All evaluated machine learning algorithms (XG, LGBM, NB, NN, SVM, RF, ST, LR) were to similar degrees able to identify patterns for classification of MC (all) against the combined group of controls and against active UC (Figure S3). Regularized LR had one of the best performances, within practical equivalence, and was chosen for classification and interpretation of model variable importances (Figure S3). The classification models show good performance in the prediction of active MC against the combined controls (AUC = 0.93). Among the top analytes for the classification, IL-6Rα, granzyme B, 4-1BB, and MMP-3 display the highest importance scores (Figure 6A). Models for the prediction of MC-HR show difficulties in separating MC-HR from the controls (AUC = 0.61) and a moderate performance in separating them from active UC (AUC = 0.75) (Figure 6A and B). However, the classification of all MC patients (active disease and in remission) against the combined control group shows good performance (AUC = 0.87) (Figure 6A). The variables used for model predictions of all MC patients show similarities with models for the individual MC groups, where CCL4, 4-1BB, MMP-3, granzyme B, and IL-6Rα display the highest importance scores (Figure 6A).

A good prediction of active MC patients from active UC was obtained (AUC = 0.91), and the classification of all patients with MC (those with active disease and in remission) from active UC shows equally good performance (AUC = 0.91) as models predicting active MC. The most important analytes in the classification models of patients with active MC against active UC were granzyme B, IL-6Rα, FasL, and IL-1β, and the top analytes for classification of all MC patients were FasL, granzyme B, BAFF, and IL-1β (Figure 6B).

## Discussion

To our knowledge, this is the first study to examine and report significant differences in the levels of granzyme B, Fas, perforin, BCMA, chitinase 3-like 1, pentraxin-3, CD163, TNF-RII, gp130, IL-6Rα, and IL-33 in patients with MC compared to patients with UC and controls. This study also corroborates previous findings by our research group of increased protein levels in MC patients of 4-1BB, APRIL, BAFF, CCL4, and CXCL8, as well as increased CCL5 gene expression, compared to controls and/or diarrhea controls.^13,21^ Additionally, our findings of increased MMP-1 in MC and MMP-3 in CC patients compared to controls, albeit MMP-1 in LC patients only compared to diarrhea controls, are in line with other groups that observed slightly increased expression of MMP-1 RNA^22^ and increased MMP-3 messenger RNA (mRNA) expression in CC patients.^23^ Furthermore, differences in colonic levels of IL-11, IL-17E, IL-19, IL-27, IL-28A, IL-31, IL-34, TNF-β, IFN-β, FasL, GM-CSF, osteopontin, aggrecan, and TNF-RI were also investigated for the first time in MC but were, in our study, with this type of Luminex analysis, non-detectable in all groups investigated.

Although the sex distribution is skewed in the present cohort, with LC active, LC-HR, and CC-HR only being represented by women, only BCMA displayed signs of confounding by sex, with higher levels being found in males. For all other analytes, levels were not associated with sex.

As mentioned previously, one advantage of fluorescence-based analysis compared to concentration-based analysis is the analysis of analytes with low levels being possible as MFI values are reported for all samples, removing the need for imputation, whereas protein concentrations sometimes are not reported if the sample falls outside of the range of standards.^14^

We have previously observed increases in CD8^+^ T cells in patients with MC.^9,10^ CD8^+^ T cells express granzyme B, perforin, and Fas and play a role in immune surveillance.^8^ There are no previous data about these analytes in MC patients. Novel findings from the present study were higher levels of granzyme B in CC compared to UC patients and increased levels of perforin in both LC and CC compared to both control groups, which support our previous observations of the increased presence of CD8^+^ cells in MC.^9,10^

Tumor necrosis factor receptor II, expressed on CD8^+^ T cells, facilitates their cytotoxic activities and also regulates activation-induced cell death.^24^ Our novel findings of increased levels of TNF-RII in MC patients compared to controls may reflect the increased presence of CD8^+^ T cells in MC patients. TNF-RI, expressed on many cell types,^24^ was found to be below the cutoff criteria in this study.

The binding of FasL to Fas causes apoptosis in Fas-expressing cells.^25^ Epithelial apoptosis in patients with CC and LC has been found to be indistinguishable compared to controls,^26,27^ whereas UC patients exhibit increased apoptosis.^25^ No previous studies of Fas and FasL levels in MC patients have been conducted. The role of Fas/FasL in MC is difficult to judge from the present findings as no significant changes in FasL were found.

CD8^+^ T cells, in addition to releasing perforin and granzymes, also degranulate CCL5 (RANTES).^20^ In accordance with a previous study by our group, CCL5 was increased in patients with active LC compared to LC-HR and, although not significant in the present study, there was a trend of increased levels compared to controls.^21^

We found more increases of CCL20 and CXCL8 protein levels in UC compared to LC and CC patients. Our previous study did not show increased mRNA levels of CCL20 among LC or CC patients while UC patients displayed elevated levels^28^ but the correlation between protein and mRNA levels is variable.^29^ CCL20 recruits T helper (Th) 17 cells^30^ which, following induction by IL-1β and IL-23, produce IL-17^31^ that induces the production of CXCL8 leading to neutrophil recruitment.^32^ The present, as well as a previous study by us,^21^ demonstrated increased levels of CXCL8 in CC and LC patients, and a study by Pearl et al. revealed UC patients with heightened signs of inflammation also showed higher levels of CXCL8.^33^ Neutrophil influx signifies active disease in CD and UC,^34^ whereas patients with LC or CC have fewer neutrophils^35^ or a complete lack of neutrophil activity.^36^ In line with these observations, the present study detected lower levels of neutrophil-recruiting chemokines in MC compared to UC.

In addition, pentraxin-3, a member of the pentraxin family together with CRP, has recently been identified as a potential biomarker for UC,^37^ which was corroborated in the present study. We have been unable to find any studies investigating pentraxin-3 levels in MC patients besides the present study.

We previously found that cytokines associated with Th1/Tc1 and Th17/Tc17 cells are prevalent in MC patients, with increased mRNA levels of IL-17 and IL-1β, although no significant differences in protein levels.^28^ Protein levels of IL-23A (p19 subunit) in our previous study were unchanged in MC patients,^28^ and although the current study examined the whole IL-23 molecule (IL-23A/p19 + IL-23B/p40), levels in all groups were below the cutoff criteria.

Additionally, TNF-α, IL-6, IL-21, as well as Th1-associated IL-12 p70 and IFN-γ and the Th2-associated cytokines IL-4- IL-5, and IL-10, failed to meet the cutoff criteria of the present study. We have previously found enhanced protein levels of TNF-α in CC and UC, IL-6 in CC and IL-21 in both CC and LC patients as well as increased mRNA levels of IL-6 and IL-21 in MC and UC patients.^28^ The disparity between our previous and current results may be due to the use of different Luminex kits from different manufacturers. We have also previously observed unchanged protein levels of IL-12 p70 and IFN-γ in MC and UC, in addition to a lack of changes in IL-4, IL-5, and IL-10,^28^ and the latter finding may indicate a lesser involvement of Th2 cells in MC.

Interleukin-33 has not previously been studied in MC patients and this study revealed that increased levels were only seen in UC patients, corroborating the previously reported increased mRNA levels in UC patients compared to patients with CD and controls.^38^

Similar to our previous findings, CCL4 was significantly increased in CC and LC compared to diarrhea controls and in UC compared to both control groups, although in the previous study, no comparisons were made to UC patients, and the controls were not divided into diarrhea and non-diarrhea controls.^21^

The current study revealed for the first time that MC patients display increased levels of chitinase 3-like 1 compared to both control groups, with levels in UC patients surpassing those in MC.

Similarly, the protein levels of the macrophage marker and scavenger receptor CD163 have not previously been studied in MC, although it has been found to be more highly expressed in IBD patients compared to controls.^39^ Here, we show that CC patients differ from LC patients in having increased levels of CD163 compared to diarrhea controls.

Matrix metalloproteinases can degrade the extracellular matrix and are regulated by tissue inhibitor of metalloproteinases (TIMPs).^22^ In line with our findings of increased levels of MMP-1 and MMP-3 in MC patients, previous studies in CC patients have demonstrated slightly increased MMP-1 expression and increased expression of TIMP-1^22^ as well as increased MMP-3 gene expression.^23^

APRIL and BAFF, involved in B-cell maturation, were previously found by us to be increased in CC and UC patients compared to diarrhea controls^13^ and this was also observed in the present study. APRIL and BAFF can both bind to BCMA, and all contribute to the survival of plasma cells.^40^ BCMA has not previously been investigated in MC patients but was in this study increased in CC compared to diarrhea controls. The highest levels of BCMA were found in UC patients, the majority of which were males, and possible signs of confounding by sex were observed for this analyte. Therefore, the association between BCMA and diagnosis should be interpreted with caution.

Similarly, co-stimulatory receptor 4-1BB has in our previous study been found to be increased in LC patients compared to non-diarrhea controls and in CC and UC patients compared to both control groups,^13^ and these findings were confirmed by the current study, with increased levels in LC, CC, and UC patients compared to both control groups.

Granzyme B, 4-1BB, and MMP-3, having high importance scores in the predictive models for classification of MC patients with active disease as well as all MC patients, against the combined control group, were also significantly increased in MC patients with active disease in the univariable analysis. IL-6Rα, being significantly decreased in the univariable analysis in MC patients compared to non-diarrhea controls, also showed importance in the LR models for prediction.

Granzyme B, being significantly increased in patients with active CC compared to patients with active UC in the univariable analysis, also showed importance in the model predictions of MC active and all MC patients compared to UC active patients. Fas Ligand was below the cutoff value for inclusion in the univariable analysis but had a high importance score in both models comparing MC patients to UC patients. BAFF had a high importance score for prediction when comparing MC and UC patients and was significantly increased in UC patients in the univariable analysis. IL-6Rα had a high importance score in the model comparing MC active and UC active patients and was increased in UC active compared to MC active patients in the univariable analysis. Although IL-1β had one of the highest importance scores in the LR models when comparing MC patients with UC patients, it was just below the cutoff criteria for inclusion in the univariable analysis, in which levels were increased solely in UC patients with active disease (data not shown).

Levels of APRIL, BCMA, CCL4, CD163, Fas, gp130, IL-6Rα, CXCL8, MMP-1, and pentraxin-3, were significantly lower in diarrhea controls compared to controls in the present study and the reasons for this are unknown. One limitation of this study is that the underlying causes of diarrhea in the diarrhea controls are largely unknown, and while there was no indication that the diarrhea controls had MCi, IBS could not be ruled out in all patients based on the available information.

Another limitation of this study is the small sample size but, on the other hand, a strength of this study is the well-defined diagnosis groups, namely the separation of non-inflamed controls into 2 groups based on the presence or absence of diarrhea, as well as the investigation of LC and CC patients with clinical symptoms but no histopathological signs of disease.

Another strength is that several analytes were investigated for the first time in patients with MC, contributing to increased knowledge about the immune response in these patients.

In conclusion, although MC patients have less severe signs of macroscopic inflammation compared to patients with IBD,^1,12^ this study shows that changes in mediators within the colonic mucosa are still present in MC. Due to different patterns of expression of the analytes in MC compared to UC, it is conceivable that the pathogenesis of these 2 diseases is unique. Significant differences between LC and CC were noted regarding the levels of MMP-1 and MMP-3, and observable, albeit insignificant, changes in LC compared to CC were noted in many of the analytes, which suggest that LC and CC are 2 distinct entities.

## Supplementary Data

Supplementary data is available at *Inflammatory Bowel Diseases* online.

izaf064_suppl_Supplementary_Figures_S1-S3

izaf064_suppl_Supplementary_Table

izaf064_suppl_Supplementary_Files

## Data Availability

The R code used in the univariable statistical analysis and multidimensional scaling (MDS) is included as Supplementary Data Files S1 and S2. The datasets generated and/or analyzed during the current study are not publicly available due to current Swedish ethical legislation and European Union GDPR Act but are available from the corresponding author on reasonable request if appropriate permits are obtained from adequate authorities.
