# Supplementary material for: Increased Colonic Levels of CD8+ Cytotoxic T lymphocyte-Associated Mediators in Patients With Microscopic Colitis
Source: Inflamm Bowel Dis. 2025 Apr 10;31(8):2231–43. doi: 10.1093/ibd/izaf064 (PMC12342803; doi:10.1093/ibd/izaf064)
Supplement: izaf064_suppl_Supplementary_Figures_S1-S3 [file izaf064_suppl_supplementary_figures_s1-s3.zip › Supplementary figure 1-3/Fig text alt text supplem materials, graphical abstract.docx]

Figure text for Supplementary Figure 1: Multidimensional scaling (MDS) analysis, similar to principal component analysis (PCA), was performed on the 22 analytes with an analyte / blank MFI ratio above the set cut-off of 5.7. The variables diagnosis, sex, and age group were investigated. The diagnosis groups were patients with collagenous colitis in histological remission (CC-HR), active collagenous colitis (CC active), controls, diarrhea controls, patients with lymphocytic colitis in histological remission (LC-HR), active lymphocytic colitis (LC active), active ulcerative colitis (UC active), and UC in remission. Patients were divided into the following five age groups: 19-41, 42-52, 54-63, 64-71, and 72-88 years.

Supplementary Figure 1 alt text: Nine multidimensional scaling (MDS) plots for the analytes included in the final statistical analysis. There are three plots per row investigating different variables, diagnosis in the first row, sex in the second, and age group in the third.

Figure text for Supplementary Figure 2: Multidimensional scaling (MDS) analysis, similar to principal component analysis (PCA), was performed on the 22 analytes with an analyte / blank MFI ratio above the set cut-off of 5.7. The variable plate (1-3) was investigated separately for each panel (1-7). Analytes included in each panel are described in detail in Table 2 in the main body of the manuscript.

Supplementary Figure 2 alt text: 21 multidimensional scaling (MDS) plots for the analytes included in the final statistical analysis. There are three plots per row investigating the variable plate (1-3) that was investigated separately for each panel (1-7).

Figure text for Supplementary Figure 3: Evaluation of machine learning algorithm strengths for classification. Performance of classification of MC (all) against controls (A) or against UC active (B) in parallel nested cross-validations with different machine learning algorithms. The upper section shows the averaged receiver operating characteristic (ROC) curve (mean area under the curve (AUC) shown in legend parentheses) of nests in nested cross-validations. The lower section shows the distribution of posteriors for the mean AUC, from the nests in nested cross-validations, for evaluation of practical equivalence. LGBM = light GBM; LR = penalised regularised logistic regressions; NB = Naïve Bayes; NN = neural network; ST = stacked model of LR, XG and NN; SVM = support vector machine; RF = random forest, and XG = XGBoosted trees.

**Supplementary Figure 3 alt text: Two plots showing the performance of the machine learning algorithms that were tested in patients with microscopic colitis compared to both control groups (A) and in patients with microscopic colitis compared to ulcerative colitis (B).**

Supplementary Table 1 alt text: A table showing the results of Spearman’s rank correlation performed to investigate the relationship between age and analyte levels.

Graphical abstract alt text: Graphical abstract showing which analytes were increased in patients with microscopic colitis compared to ulcerative colitis, similar in microscopic colitis and ulcerative colitis, increased in both these groups compared to controls, decreased in microscopic colitis compared to controls, and increased in ulcerative colitis compared to microscopic colitis.
