# Supplementary figures and images for: Increased Colonic Levels of CD8+ Cytotoxic T lymphocyte-Associated Mediators in Patients With Microscopic Colitis
Source: Inflamm Bowel Dis. 2025 Apr 10;31(8):2231–43. doi: 10.1093/ibd/izaf064 (PMC12342803; doi:10.1093/ibd/izaf064)

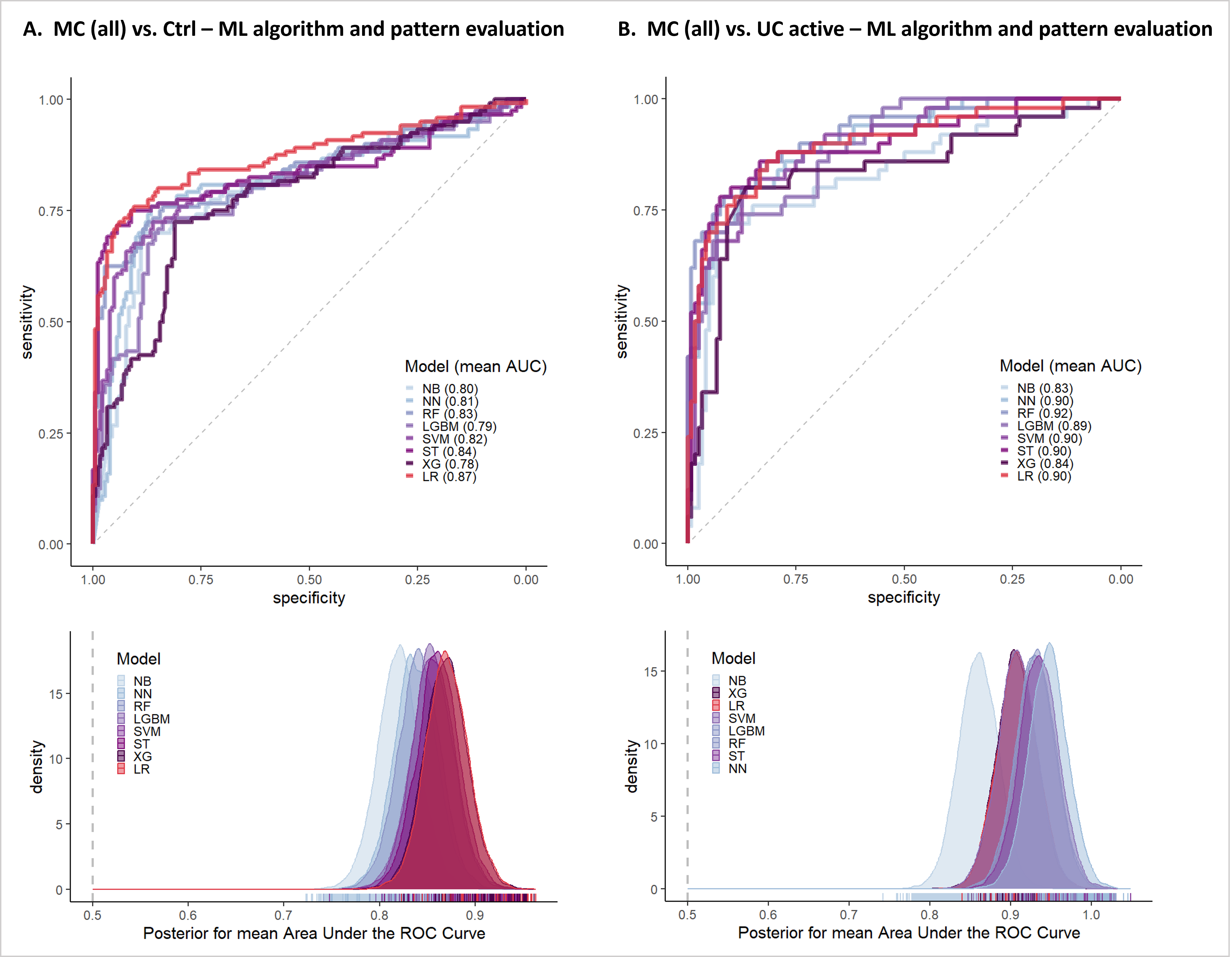

Supplement: izaf064_suppl_Supplementary_Figures_S1-S3 [file izaf064_suppl_supplementary_figures_s1-s3.zip › Supplementary figure 1-3/Supplementary Figure 3 (new colours).tif]
