# Supplementary material for: Increased Colonic Levels of CD8+ Cytotoxic T lymphocyte-Associated Mediators in Patients With Microscopic Colitis
Source: Inflamm Bowel Dis. 2025 Apr 10;31(8):2231–43. doi: 10.1093/ibd/izaf064 (PMC12342803; doi:10.1093/ibd/izaf064)
Supplement: izaf064_suppl_Supplementary_Table [file izaf064_suppl_supplementary_table.pdf]

Supplementary Table 1. Spearman's rank correlation performed to investigate the relationship between age and analyte levels.

**Analyte**	**Spearman's rank correlation rho**	**P-value**
4-1BB	-0.061	0.45
APRIL	-0.029	0.72
BAFF	-0.13	0.098
BCMA	-0.0036	0.96
CCL4	0.0098	0.90
CCL5	0.074	0.36
CXCL8	0.0046	0.95
CCL20	-0.13	0.11
CD163	0.056	0.49
Chitinase 3-like 1	-0.014	0.86
Fas	-0.0093	0.91
Granzyme A	0.089	0.29
Granzyme B	0.088	0.28
Gp130	-0.13	0.096
IL-6Rα	-0.090	0.27
IL-33	-0.23	0.0046
Perforin	-0.12	0.22
Pentraxin-3	-0.046	0.57
MMP-1	-0.039	0.63
MMP-2	0.084	0.34
MMP-3	-0.072	0.37
TNF-RII	-0.16	0.059
